# Supplementary material for: The RNA-Binding Protein HuD Regulates Alternative Splicing and Alternative Polyadenylation in the Mouse Neocortex
Source: Molecules. 2021 May 11;26(10):2836. doi: 10.3390/molecules26102836 (PMC8151252; doi:10.3390/molecules26102836)
Supplement: Supplementary file 1 [file molecules-26-02836-s001.zip › Rev2_Supplementary tables 1-8 and Fig S1_Molecules/Figure S1.pdf]

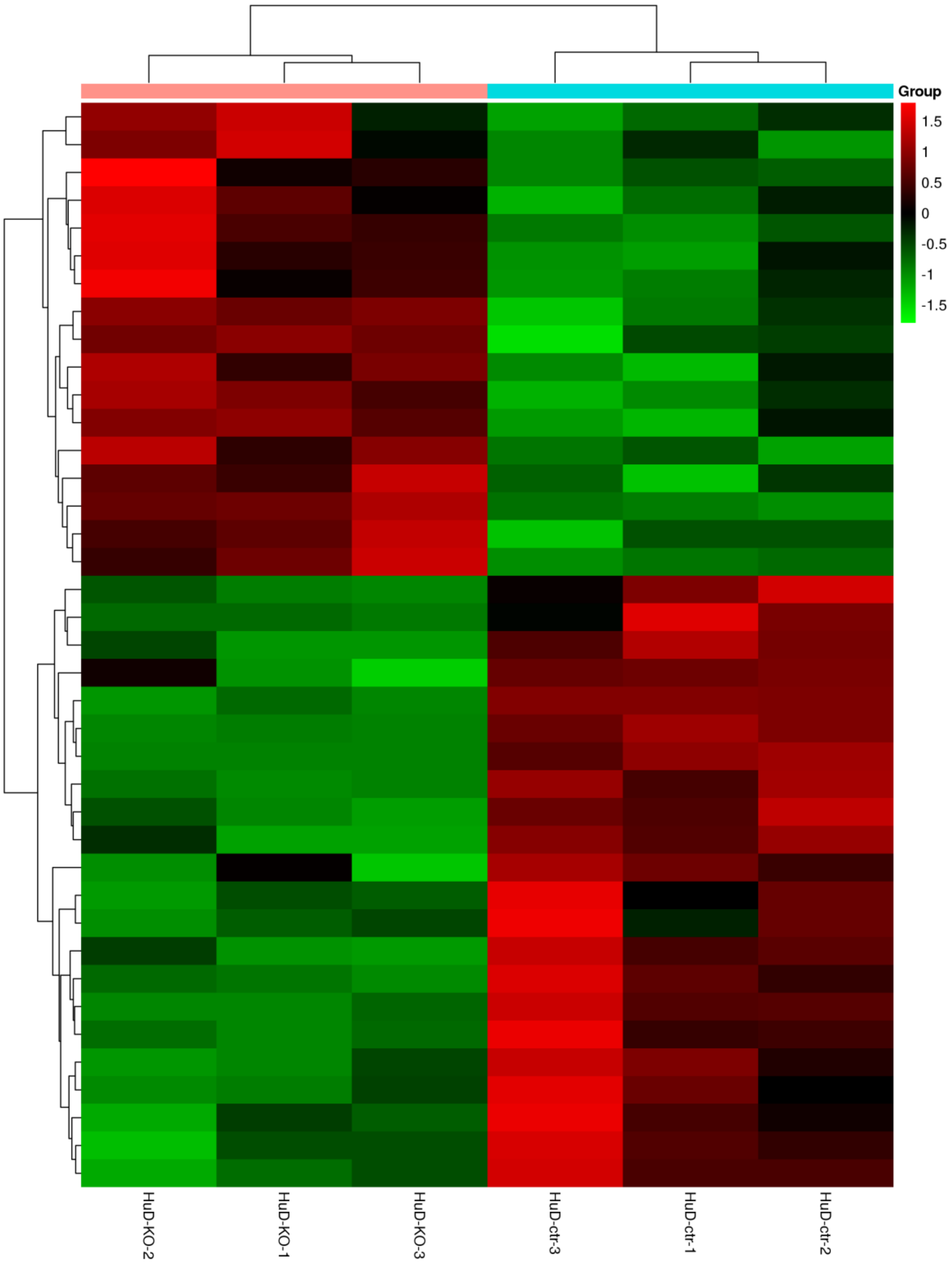

Figure S1. Unsupervised hierarchical clustering analysis of differentially expressed mRNAs in the neocortex of HuD KO and control mice. The heatmap was obtained using genes that were significantly differentially expressed. Each row represents a gene and each column represents a sample. The color scale represents the relative expression level (log2 scaled FPKM), red indicates higher values, and green indicates lower values.
